# Comment on “Differential effects of pre-procedural atorvastatin versus rosuvastatin on hematologic and inflammatory markers in patients with ST-elevation myocardial infarction undergoing primary percutaneous coronary intervention: A randomized controlled trial”

**DOI:** 10.1016/j.ahjo.2026.100974

**Published:** 2026-10-16

**Authors:** S. Dhanalakshmi, K.S. Sridevi Sangeetha, D. Ganesh Kumar, R. Nandhini

**Affiliations:** a Meenakshi College of Pharmacy, Meenakshi Academy of Higher Education and Research, India; b Meenakshi College of Allied Health Sciences, Meenakshi Medical College Hospital & Research Institute, Meenakshi Academy of Higher Education and Research, India; c Department of Pharmacology, Meenakshi Medical College Hospital & Research Institute, Meenakshi Academy of Higher Education and Research, Enathur, Kanchipuram, Tamil Nadu, 631552, India

**Keywords:** Atorvastatin, Rosuvastatin, ST-elevation myocardial infarction, Percutaneous coronary intervention, Neutrophil-to-lymphocyte ratio, Platelet distribution width, Inflammatory markers, Hematologic markers, High-intensity statins, Randomized controlled trial

Dear Editor,

Khameneh Bagheri et al. [1], compared pre-procedural atorvastatin 80 mg with rosuvastatin 40 mg in 200 patients with STEMI, reporting a lower neutrophil-to-lymphocyte ratio with atorvastatin and a higher platelet distribution width with rosuvastatin. A randomized comparison of two high-intensity statins in this setting is worth having. The reported numbers do not survive checking.

The abstract does not match the results. For the 48-hour ratio the abstract gives medians with interquartile ranges of 5.1 [3.2 to 8.1] against 7.8 [4.9 to 10.2] at *p* = 0.003. The results give 4.93 ± 3.88 against 7.23 ± 5.46 at *p* = 0.001, and the analysis note says every *p*-value came from an independent *t*-test. No median comparison appears anywhere, so the abstract's central result has no source. The 24-hour width is given in the abstract as 14.7 ± 1.5 for atorvastatin when the result is 14.91 ± 1.26.

The baseline percentages use the wrong denominator: 61 of 102 men in the rosuvastatin arm is 59.8%, reported as 54.5%, which is 61 of 112, the share of all men in the study [2]. The same applies to the other three cells, and the reported pairs sum to 101.1% and 98.9%.

Several baseline *p*-values cannot be reproduced. Diabetes is 13 of 102 against 16 of 98 at a stated *p* = 0.10, and addiction carries the identical counts at a stated *p* = 0.54. One pair of counts cannot yield two *p*-values, and both recompute to 0.47 by chi-squared, 0.55 by Fisher's exact test. Hypertension at 68.6% against 83.7% is reported at *p* = 0.14 and recomputes to 0.013, so an imbalance has been presented as balance [3]. LDL cholesterol recomputes to 0.030 against a reported 0.06.

Baseline white cell count was 10.19 ± 2.81 against 8.62 ± 1.80, which gives p below 0.0001, yet the groups are described as balanced apart from HDL [4]. The ratio derives from the differential count, so a difference of that size bears on the headline finding, and no baseline-adjusted analysis is given. At 48 h it reverses direction, with a standard deviation of 9.72 in the atorvastatin arm against 1.80 at baseline. Ratios whose standard deviations run at 76% to 85% of their means were compared by *t*-test throughout.

The nephropathy model is inconsistent with itself. An odds ratio of 0.416 with an interval of 0.28 to 0.73 implies a log-scale standard error near 0.24, so p near 0.0003, not the stated 0.033. An area under the curve of 0.318 is reported as a strong association, but anything below 0.5 means the direction is inverted; the correct value is 0.682. Both rest on seven events, and this analysis appears in neither the methods nor the results.

Two citations do not support their claims. The source given for width as a marker of platelet activation is Beyan and colleagues, who found no correlation with platelet aggregation responses. The sentence claiming agreement with the authors' own primary results cites the HORIZONS-AMI substudy instead. A mean TIMI value of 20.42 also lies outside that score range.

## CRediT authorship contribution statement

**S. Dhanalakshmi:** Visualization, Writing – original draft, Writing – review & editing. **K.S. Sridevi Sangeetha:** Conceptualization, Data curation, Formal analysis. **D. Ganesh Kumar:** Visualization, Writing – original draft, Writing – review & editing. **R. Nandhini:** Visualization, Writing – original draft, Writing – review & editing.

## Informed consent

Not applicable.

## Ethical approval

Not applicable (commentary on a previously published article).

## Declaration of Generative AI and AI-assisted technologies in the writing process

Generative AI tools were used only for language refinement and formatting assistance. All scientific interpretation, critical appraisal, and manuscript preparation were performed independently by the authors.

## Funding

None.

## Declaration of competing interest

The authors declare that there are no conflicts of interest.

## Data Availability

No new data were generated or analyzed in this study.
